# BCAT1 binds the RNA-binding protein ZNF423 to activate autophagy via the IRE1-XBP-1-RIDD axis in hypoxic PASMCs

**DOI:** 10.1038/s41419-020-02930-y

**Published:** 2020-09-16

**Authors:** Wei Xin, Min Zhang, Yang Yu, Songlin Li, Cui Ma, Junting Zhang, Yuan Jiang, Yiying Li, Xiaodong Zheng, Lixin Zhang, Xijuan Zhao, Xuzhong Pei, Daling Zhu

**Affiliations:** 1 College of Pharmacy, Harbin Medical University, Harbin, 150081 P.R. China; 2 Central Laboratory of Harbin Medical University (Daqing), Daqing, 163319 P.R. China; 3 Division of Cardiology and Hubei Key Laboratory of Genetics and Molecular Mechanisms of Cardiological Disorders, Tongji Hospital, Tongji Medical College, Huazhong University of Science and Technology, Wuhan, 430030 P.R. China; 4 College of Pharmacy, Harbin University of Commerce, Harbin, 150076 P.R. China; 5 College of Medical Laboratory Science and Technology, Harbin Medical University (Daqing), Daqing, 163319 P.R. China; 6 Department of Genetic and Cell Biology, Harbin Medical University (Daqing), Daqing, 163319 P.R. China; 7 State Province Key Laboratories of Biomedicine-Pharmaceutics of China, Daqing, 163319 P.R. China; 8 Key Laboratory of Cardiovascular Medicine Research, Ministry of Education, Harbin Medical University, Harbin, 150081 P.R. China

**Keywords:** Autophagy, Post-translational modifications

## Abstract

Abnormal functional changes in pulmonary artery smooth muscle cells are the main causes of many lung diseases. Among, autophagy plays a crucial role. However, the specific molecular regulatory mechanism of autophagy in PASMCs remains unclear. Here, we first demonstrate that BCAT1 played a key role in the autophagy of hypoxic PASMCs and hypoxic model rats. BCAT1-induced activation and accumulation of the autophagy signaling proteins BECN1 and Atg5 by the endoplasmic reticulum (ER) stress pathway. Interestingly, we discovered that BCAT1 bound IRE1 on the ER to activate expression of its downstream pathway XBP-1-RIDD axis to activate autophagy. More importantly, we identified an RNA-binding protein, zinc finger protein 423, which promoted autophagy by binding adenylate/uridylate (AU)-rich elements in the BCAT1 mRNA 3′-untranslated region. Overall, our results identify BCAT1 as a potential therapeutic target for the clinical treatment of lung diseases and reveal a novel posttranscriptional regulatory mechanism and signaling pathway in hypoxia-induced PASMC autophagy.

## Introduction

Smooth muscle cells in the vessel wall are the main cell type essential for the structural and functional integrity of blood vessels. Abnormal functional changes in pulmonary artery smooth muscle cells (PASMCs) are the main cause of many lung diseases. At present, lung diseases, including pulmonary hypertension, pulmonary fibrosis, chronic obstructive pulmonary disease, and even lung cancer, are a type of disease with extremely high mortality^1–3^. Although different diseases have different characteristics and pathogenesis, PASMC dysfunction plays an important role in the pathogenesis of various lung diseases^4–6^. Therefore, it is extremely important to further study the functions and regulation mechanisms of PASMCs, and the need to find new therapeutic targets is urgent.

The homeostasis of oxygen is an important factor in maintaining normal lung structure and function^7^. In fact, hypoxia is a key cause of lung diseases such as pulmonary hypertension and pulmonary fibrosis. Increasing evidence shows that hypoxia has a significant effect on the behavior of many kinds of tumors, including non-small cell lung cancer (NSCLC)^8–10^. Hypoxia occurs in early NSCLC and is related to the increased expression of osteopontin and carbonic anhydrase IX^10^. Importantly, hypoxia is an important pathogenic factor for PASMC proliferation, migration, apoptosis, and pyroptosis^11^. Hypoxia led to the upregulation of mitofusin 1, which maintains mitochondrial homeostasis through mir-125a, and was shown to play an important role in promoting PASMCs^12^. We reported that programmed death ligand 1 triggered pyroptosis and pulmonary fibrosis in hypoxic PASMCs^9^. Therefore, study of the molecular mechanism of hypoxia in PASMCs may provide a new target for the diagnosis and treatment of many lung diseases.

Autophagy is a programmed intracellular homeostatic process that plays an important role in maintaining the balance between protein synthesis and degradation in cells^13^. Autophagy occurs in many lung diseases, such as chronic obstructive pulmonary disease, idiopathic pulmonary fibrosis, and pulmonary hypertension^14–16^. Our previous studies confirmed that acetylated cyclophilin A is an important mediator of hypoxia-induced autophagy and pulmonary angiogenesis, indicating that hypoxia can induce autophagy in pulmonary artery endothelial cells (PAECs)^17^. However, the role of hypoxia in PASMC autophagy remains unclear.

Branched-chain amino transferases (BCAT) are enzymes that carry out the reversible transamination of all three branched-chain amino acids (BCAAs) to branched-chain α-ketoacids and glutamate. There are two BCAT isoenzymes, cytosolic BCAT1 and mitochondrial BCAT2^18^. The differential expression of BCAT1 plays a key role in many diseases. Overexpression of BCAT1-induced proliferation, migration and invasion of cells in nasopharyngeal carcinoma^19^. BCAT1-promoted cell proliferation through amino acid catabolism in human glioma carrying wild-type isocitrate dehydrogenase 1^20^. However, the relationship between BCAT1 and autophagy is not clear. For this reason, BCAT1 may serve as a regulatory factor that participates in the regulation of PASMC autophagy.

The posttranscriptional regulation of specifically targeted mRNA is crucial for maintaining proper cell physiology and alters the cellular response to stress, autophagy, proliferation, apoptosis, and immune stimulation^21–23^. However, the posttranscriptional regulatory mechanism of autophagy in PASMCs remains unknown. (Adenylate/uridylate) (AU)-rich elements (AREs) in 3′-untranslated regions (UTRs) can provide binding sites for transcriptionally active RNA-binding proteins (RBPs), thereby regulating the stability and translation of transcripts^24^. Interestingly, certain types of zinc finger proteins are RBPs that regulate RNA metabolism^25^. ZNF423, which is required for cerebellar development^26–28^, is involved in the development of B lymphoblastic leukemia^29,30^. Silencing of ZNF423 has been reported to significantly inhibit cell proliferation and invasion of the cholangiocarcinoma cell line^31^. However, it is unclear whether ZNF423 can be used as an RBP to activate autophagy in PASMCs through posttranscriptional regulatory mechanisms.

In this study, we found for the first time that hypoxia induced the high expression of BCAT1, which bound IRE1 in the endoplasmic reticulum (ER) to activate the ERs pathway and the pathway downstream of IRE1, finally inducing autophagy. We also identified that ZNF423 is involved in increased BCAT1 expression through its binding of AREs in the 3′-UTR of BCAT1 mRNA for the first time. Our results indicate a novel posttranscriptional regulatory mechanism and signaling pathway in PASMC autophagy.

## Results

### Hypoxia upregulates the expression of BCAT1

**Fig. 1:**
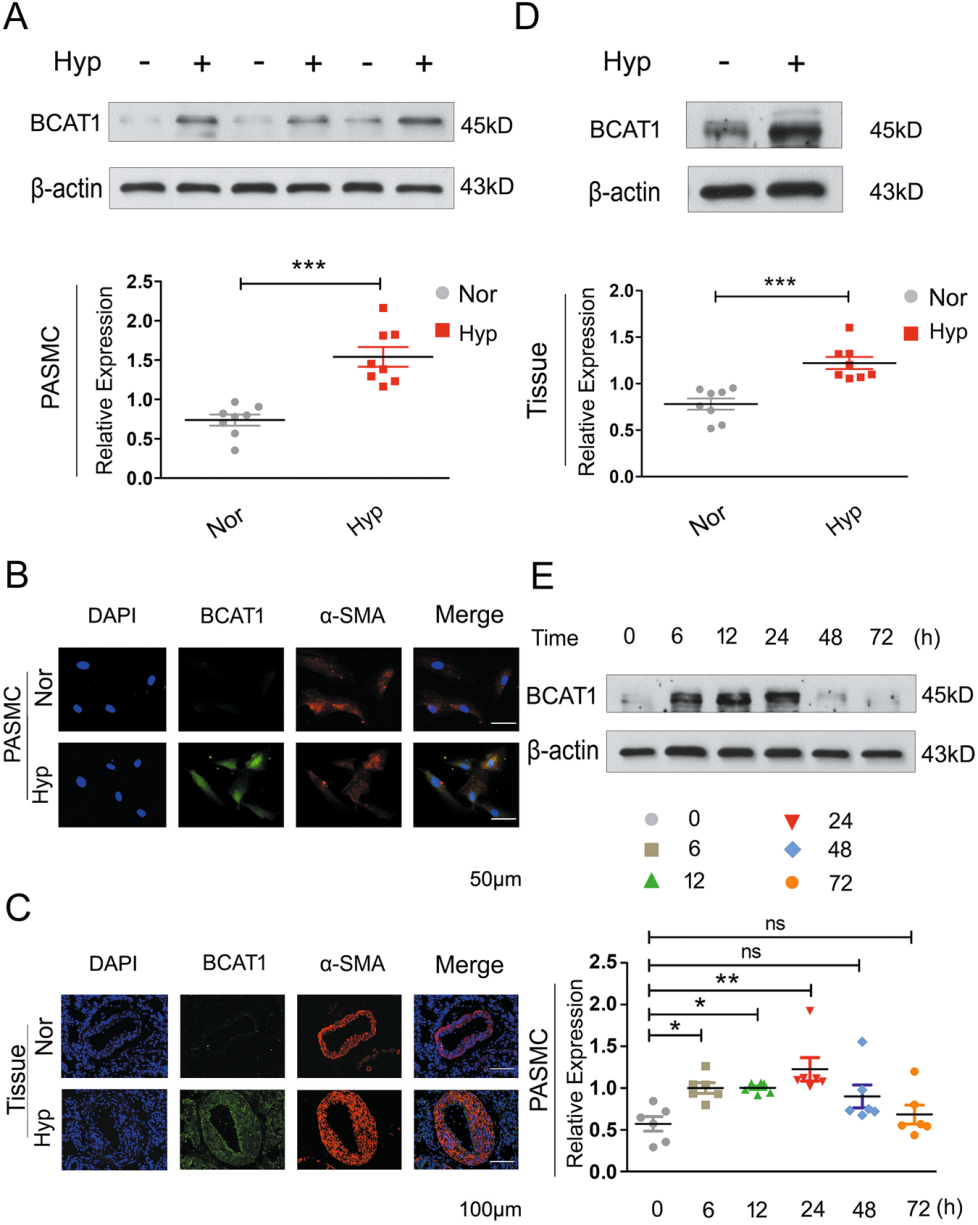
Hypoxia resultes in the increased expression of BCAT1. **a** Western blot analysis of BCAT1 expression in hypoxic PASMCs (*n* = 8). **b** Subcellular distribution of BCAT1 in PASMCs determined by immunofluorescence analysis. Scale bars: 50 μm (*n* = 3). **c** The cellular expression of BCAT1 in the smooth muscle layer of lung tissues from hypoxic model rats determined by immunofluorescence staining analysis. Scale bar = 100 μm (*n* = 3). **d** BCAT1 protein levels in pulmonary arterial tissues of hypoxia model rats (*n* = 8). **e** Time course of BCAT1 expression of PASMCs at 0, 6, 12, 24, 48, and 72 h after hypoxia treatment (*n* = 6). Nor normoxia, Hyp hypoxia, Mct monocrotaline. Statistical analysis was performed with one-way ANOVA or the Student’s *t* test. All values are presented as the mean ± SEM. *p < 0.05; **p < 0.01; ***p < 0.001; ns not significant.

First, to confirm the expression of BCAT1 in PASMCs, PASMCs were maintained under hypoxia for 24 h. BCAT1 expression was highly upregulated in the hypoxic group compared with the normal group (Fig. 1a). Coimmunostaining for BCAT1 with α-SMA, a nonspecific marker of smooth muscle cells, showed that BCAT1 was substantially upregulated in hypoxic PASMCs (Fig. 1b). Interestingly, we found that BCAT1 expression was markedly increased in hypoxic PASMCs in a time-dependent manner and peaked at 24 h after hypoxia exposure (Fig. 1e). We further explored BCAT1 expression in hypoxic model rats to confirm these results in vivo. We verified the hypoxia model rats successfully through two types of in vitro hypoxia model experiments (Fig. S1) and found that BCAT1 expression was upregulated in pulmonary arterial tissues of rats exposed to hypoxia for 21 days (Fig. 1c, d) and a BCAT1 inhibitor, gabapentin, could significantly improve the relevant hypoxia indicators of hypoxia model rats (Fig. S1). This result is consistent with the results obtained in vitro.

There are two BCAT isoenzymes^18^. To determine whether BCAT1 was the isoenzyme mainly affected by hypoxia, BCAT2 expression was also tested. Under hypoxia, there was no significant change in the expression of BCAT2 (Fig. S2a). Finally, we also examined the expression of BCAT1 in PAECs and found no significant difference in expression between the hypoxia group and the control group (Fig. S2b). These results indicated that the expression of BCAT1 was upregulated in hypoxia-induced PASMCs and hypoxic model rats.

### Upregulation of BCAT1 expression by hypoxia leads to PASMCs autophagy

**Fig. 2:**
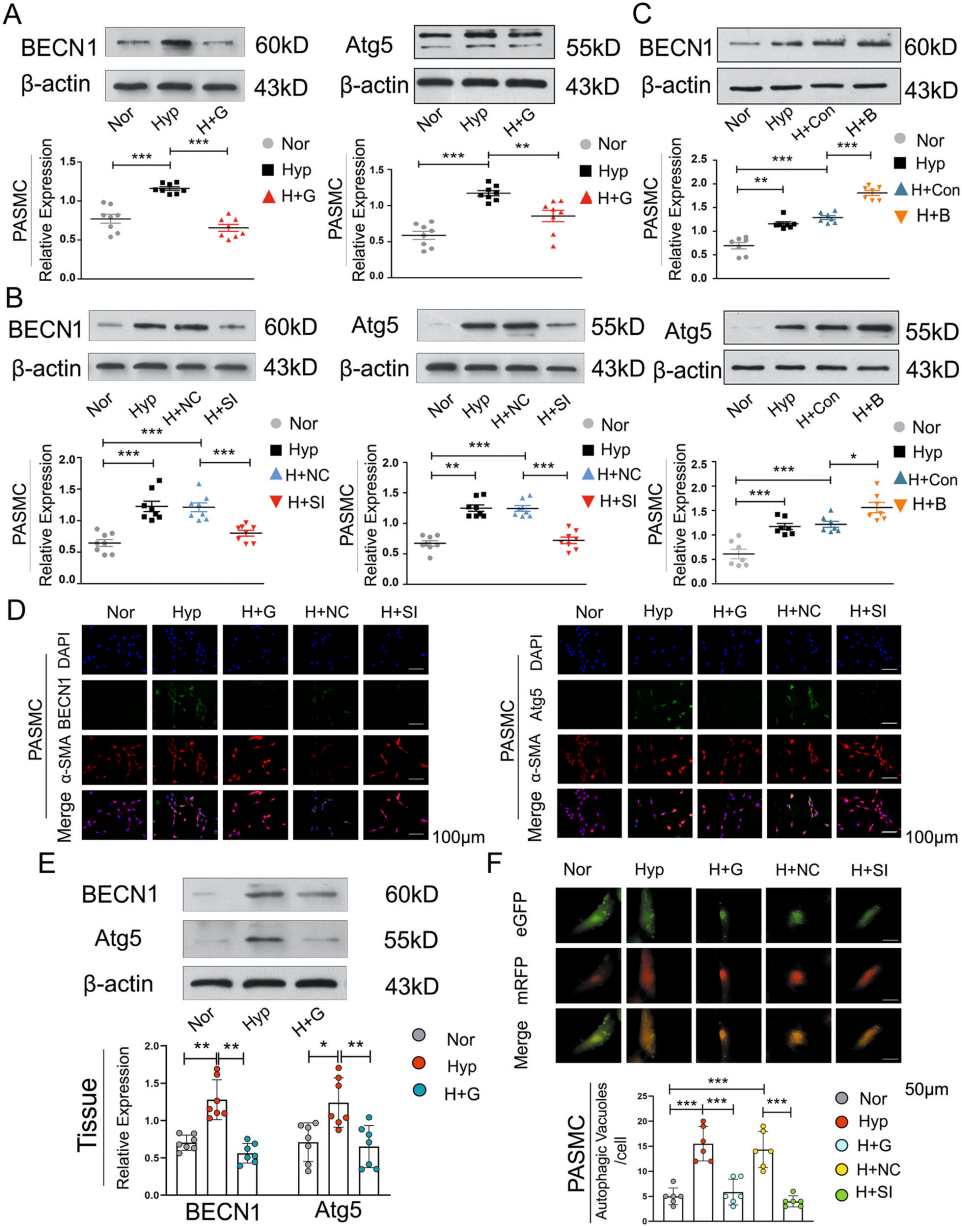
Upregulation of BCAT1 expression induced by hypoxia leads to PASMC autophagy. **a** Western blot analysis of BECN1 and Atg5 protein expression in PASMCs treated with the inhibitor gabapentin (20 µM) (*n* = 8). **b** Western blot analysis of BECN1 and Atg5 protein expression in PASMCs transfected with BCAT1 siRNA or BCAT1 plasmid (*n* = 8). **c**, **d** Immunofluorescence staining for BECN1 and Atg5 in PASMCs. BECN1 and Atg5 (green), α-SMA (red), and DAPI (blue). Scale bar = 50 μm (*n* = 3). **e** Western blot analysis of BECN1 and Atg5 expression in the pulmonary arterial tissues of hypoxia model rats treated with gabapentin (*n* = 7). **f** Measurement of autophagic flux in PASMCs transfected with eGFP-mRFP-LC3 plasmid and exposed under NOR or HYP for 24 h treated with BCAT1 siRNA or the BCAT1 inhibitor gabapentin. Yellow and red dots indicate autolysosomes and autophagosomes, respectively. Scale bar = 50 μm (*n* = 6). Nor normoxia, Hyp hypoxia, Mct monocrotaline, H + G hypoxia plus gabapentin, M + G monocrotaline plus gabapentin, H + NC hypoxia plus control siRNA, H + SI hypoxia plus BCAT1 siRNA, H + Con hypoxia plus control vector, H + B hypoxia plus BCAT1 plasmid. Statistical analysis was performed with one-way ANOVA. All values are presented as the mean ± SEM. *p < 0.05; **p < 0.01; ***p < 0.001.

Next, we explored the role of BCAT1 in the regulation of autophagy in hypoxic PASMCs. PASMCs were treated with gabapentin and BCAT1-specific small interfering RNA (siRNA) to knock down the expression of BCAT1. The autophagy-related indicators beclin-1 (BECN1) and Atg5 were detected by Western blot and immunofluorescence analyses. As shown in (Figs. 2a, b, d and S5a), the autophagy-associated accumulation of BECN1 and Atg5 was markedly increased in PASMCs under hypoxia, and the expression of autophagy-related proteins decreased with the knockout and inhibition of BCAT1 compared with that in the normal group. In addition, Western blotting was used to detect the expression of autophagy-related indicators in the pulmonary arterial tissue of model rats treated with a BCAT1 inhibitor, and the results were consistent with those at the cellular level (Fig. 2e). Subsequently, PASMCs were transfected with BCAT1 overexpression plasmid to overexpress BCAT1. The results showed that expression of the autophagy markers BECN1 and Atg5 was significantly increased when BCAT1 was overexpressed under hypoxia (Figs. 2c and S5e). To further confirm BCAT1-induced autophagy in hypoxic PASMCs, we administered eGFP-mRFP-LC3 plasmid as described previously^32^. After BCAT1 was inhibited and knocked out, there was a significant reduction in yellow and red punctate fluorescence, respectively, indicating autolysosomes and autophagosomes, respectively (Fig. 2f). Finally, as autophagy is a dynamic process, we applied bafilomycin A1 (Baf) to inhibit the fusion of autophagic vacuoles and lysosomes to detect the occurrence of autophagic flux. As a result, the accumulation of LC3B-II was reduced by the addition of the BCAT1 inhibitor (Fig. S3a). Taken together, these results indicate that BCAT1 regulated autophagy in hypoxic PASMCs.

### BCAT1 regulates autophagy through the ER stress pathway

**Fig. 3:**
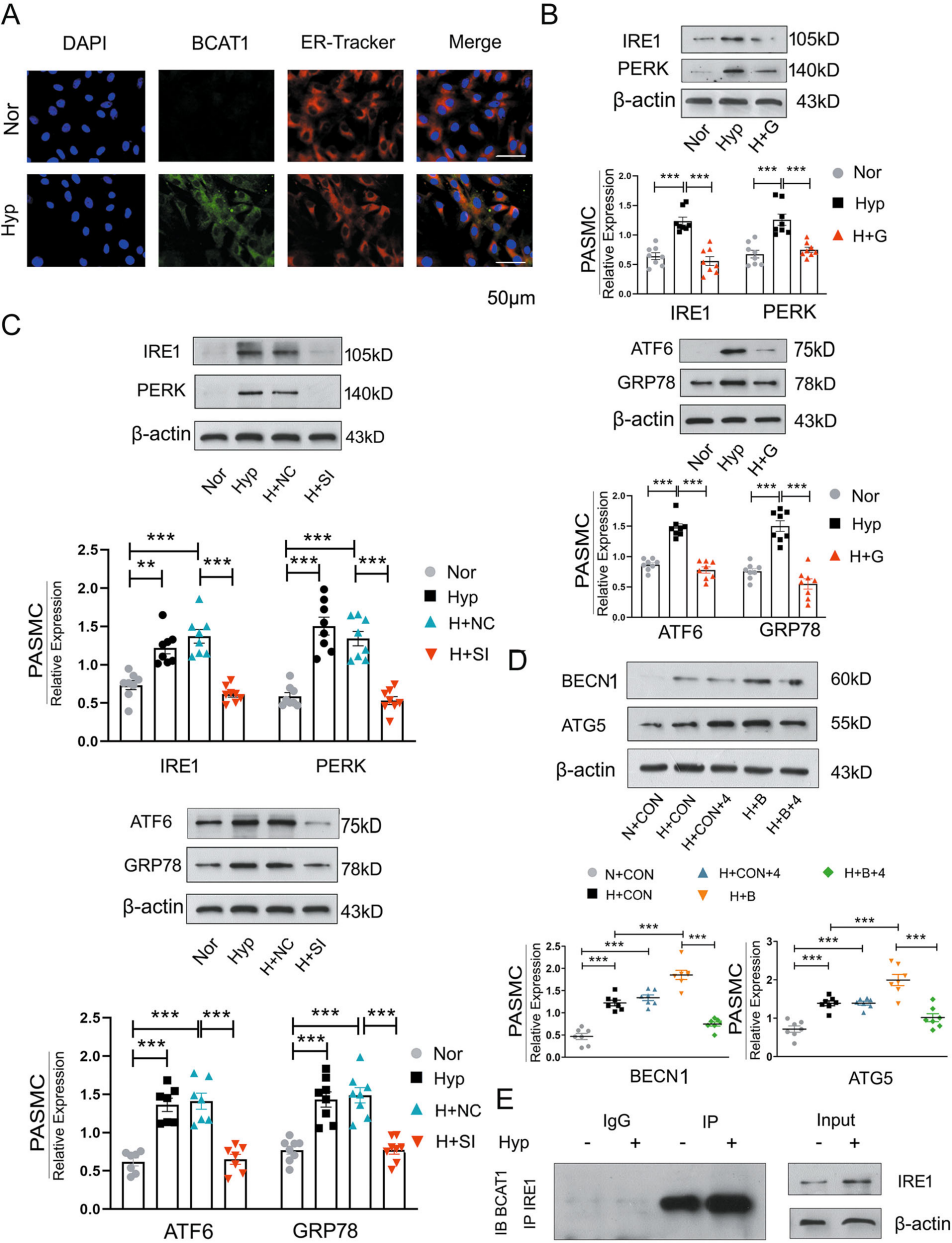
BCAT1 regulates autophagy through the endoplasmic reticulum stress pathway. **a** Expression of BCAT1 and ER-Tracker Red staining in PASMCs exposed to NOR or HYP for 24 h. Scale bar = 50 μm (*n* = 3). **b** Western blot analysis of PERK, IRE1, ATF6, and GRP78 protein expression in the ERs pathway in PASMCs treated with gabapentin (*n* = 8). **c** Western blot analysis of IRE1, PERK, ATF6, and GRP78 expression in PASMCs transfected with BCAT1 siRNA (*n* = 8). **d** Western blot analysis of BECN1 and Atg5 in PASMCs treated with the ERs pathway inhibitor 4-PBA and BCAT1 plasmid (*n* = 8). **e** Coimmunoprecipitation of the whole-cell lysates of PASMCs exposed to normoxia or hypoxia for 24 h with anti-IRE1, followed by probing with anti-BCAT1 (*n* = 3). Nor normoxia, Hyp hypoxia, H + G hypoxia plus gabapentin, H + NC hypoxia plus control siRNA, H + SI hypoxia plus BCAT1 siRNA, N + Con normoxia plus control vector, H + Con hypoxia plus control vector, H + B hypoxia plus BCAT1 plasmid, H + Con+4 hypoxia plus control vector plus 4-phenylbutyric acid, H + B + 4 hypoxia plus BCAT1 plasmid plus 4-phenylbutyric acid, IP immunoprecipitation, IB immunoblotting. Statistical analysis was performed with one-way ANOVA. All values are presented as the mean ± SEM. **p < 0.01; ***p < 0.001.

We next attempted to gain insights into the molecular mechanisms by which BCAT1 regulates autophagy under hypoxia. Although many studies have proven the relationship between autophagy and ERs^33–35^, these reports were mainly focused on the endothelium^36^. The role of BCAT1 in the smooth muscle layer has not been explored. After the expression of BCAT1 and staining with ER-Tracker Red (a red fluorescent probe indicating the ER), immunofluorescence analyses of PASMCs showed that hypoxia upregulated the expression of BCAT1 in the ER (Fig. 3a).

We further detected the expression of the marker proteins PERK, IRE1, ATF6, and GRP78 in three branches of the ERs pathway by western blotting after the treatment of PASMCs with the BCAT1 inhibitor and BCAT1 siRNA-mediated interference in PASMCs. We found that inhibition of BCAT1 could significantly reduce the increase in ERs pathway marker proteins caused by hypoxia compared with their expression in the control group (Fig. 3b, c). This result confirmed that BCAT1 induced ERs pathway activation in hypoxic PASMCs. We also used a restorative experiment to prove these findings. We overexpressed BCAT1 in PASMCs and treated the cells with the ERs pathway inhibitor 4-phenylbutyric acid (4-PBA). The results showed that overexpression of BCAT1 could significantly increase the expression of BECN1 and Atg5, while 4-PBA could significantly inhibit this phenomenon (Fig. 3d).

In addition, we investigated which pathway plays a key role in PASMC autophagy by coimmunoprecipitation of BCAT1 and ERs marker proteins. The results showed binding between BCAT1 and only IRE1, and the expression of IRE1 was increased under hypoxia (Fig. 3e and Fig. S4a). The results herein suggested that BCAT1 regulated autophagy in hypoxic PASMCs by binding IRE1 to activate the ERs pathway.

### BCAT1-regulates autophagy by activating ER stress via the IRE1-XBP1-RIDD axis during hypoxia

**Fig. 4:**
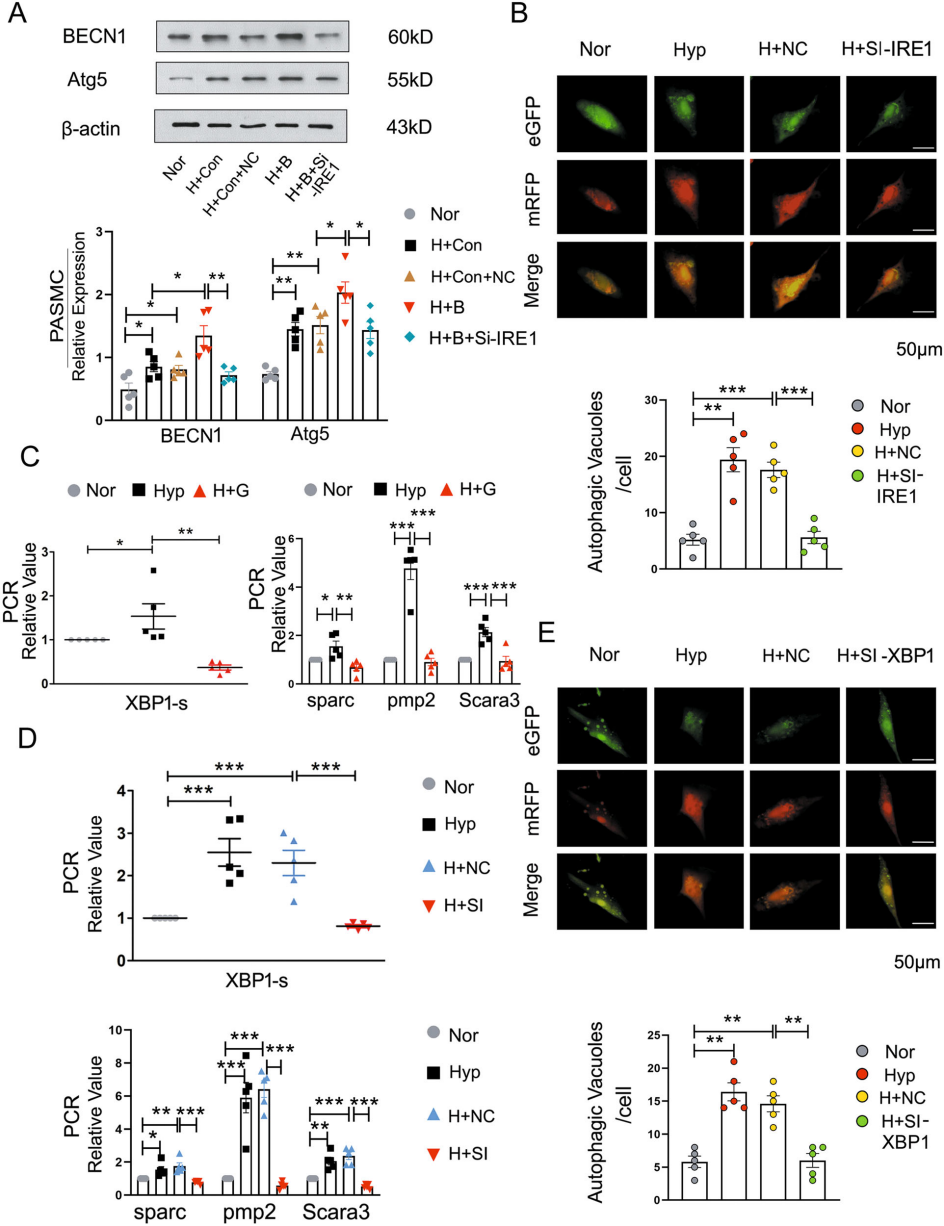
BCAT1 regulates autophagy during hypoxia by activating ERs via the IRE1-XBP1-RIDD axis. **a** Western blot analysis of BECN1 and Atg5 in PASMCs cotransfected with BCAT1 and IRE1 siRNA (*n* = 5). **b** Autophagic flux was monitored in PASMCs cotransfected with eGFP-mRFP-LC3 plasmid and control siRNA or IRE1 siRNA that were then exposed to HYP for 24 h. Scale bar = 50 μm (*n* = 3). **c**, **d** RT-PCR analysis of the mRNA levels of XBP1-s, sparc, pmp2, and Scara3 with rat β-actin serving as the standard (*n* = 5). **e** The formation of autophagosomes was detected, and autophagic activity was estimated in cells in which the expression of XBP1 was knocked down with XBP1 siRNA under HYP for 24 h. Scale bar = 50 µm (*n* = 5). Nor normoxia, Hyp hypoxia, H + G hypoxia plus gabapentin, H + NC hypoxia plus control siRNA, H + SI hypoxia plus BCAT1 siRNA, H + SI-IRE1 hypoxia plus IRE1 siRNA, H + SI-XBP1 hypoxia plus XBP1 siRNA, H + Con hypoxia plus control vector, H + B hypoxia plus BCAT1 plasmid, H + Con+NC hypoxia plus control vector plus control siRNA, H + B + Si-IRE hypoxia plus BCAT1 plasmid plus IRE1 siRNA. Statistical analysis was performed with one-way ANOVA. All values are presented as the mean ± SEM. *p < 0.05; **p < 0.01; ***p < 0.001.

To investigate the molecular mechanism by which BCAT1 binds IRE1 during hypoxia, PASMCs cotransfected with BCAT1 overexpression plasmid and IRE1 siRNA were used to detect autophagy-related protein expression. Our results showed that interference with IRE1 could significantly reverse the increased expression of autophagy-related proteins induced by overexpression of BCAT1, which indicated that the combined effect of BCAT1 and IRE1 caused by hypoxia was related to the activation of PASMC autophagy (Fig. 4a and Fig. S5b). At the same time, the eGFP-mRFP-LC3 plasmid and IRE1 siRNA were cotransfected in PASMCs to detect autophagic flux, and the cells were treated with hypoxia for 24 h. The results showed that autophagy was significantly decreased after IRE1 interference (Fig. 4b).

Interestingly, IRE1 is a component of a branch of the unfolded protein response (UPR)^37^. Its downstream pathway is related to inflammatory signal transduction through XBP1 splicing and regulated IRE1 α-dependent decay (RIDD), an innate immune function to resist potential toxicity and nucleic acids in the cytoplasm that affects innate immunity^38^. However, the role of the IRE1 downstream pathway in PASMC autophagy has not been reported. Hence, we interfered with BCAT1 with gabapentin or BCAT1 siRNA and detected the gene expression of the IRE1 downstream pathway protein XBP1 and the RIDD pathway markers XBP1-s, sparc, pmp2 and Scara3 by reverse transcription polymerase chain reaction (RT-PCR). Compared with their expression in the control group, the expression of related pathway mRNAs was reduced (Fig. 4c, d). In addition, cotransfection of PASMCs with eGFP-mRFP-LC3 plasmid and XBP1 siRNA was used to detect the formation of autophagosomes in cells cultured under hypoxia for 24 h. The results identified that autophagosomes were reduced, confirming that BCAT1 affected autophagy in hypoxic PASMCs via the XBP1 pathway (Fig. 4e). Finally, PASMC transfection with XBP1 siRNA downregulated expression of the BECN1 and Atg5 proteins (Figs. S3b and S5c). These results explicitly confirmed that BCAT1-induced autophagy in PASMCs under hypoxic conditions through the IRE1-XBP1-RIDD axis.

### Hypoxia leads to the transfer of ZNF423 to the cytoplasm and its binding with BCAT1 to promote autophagy activity

**Fig. 5:**
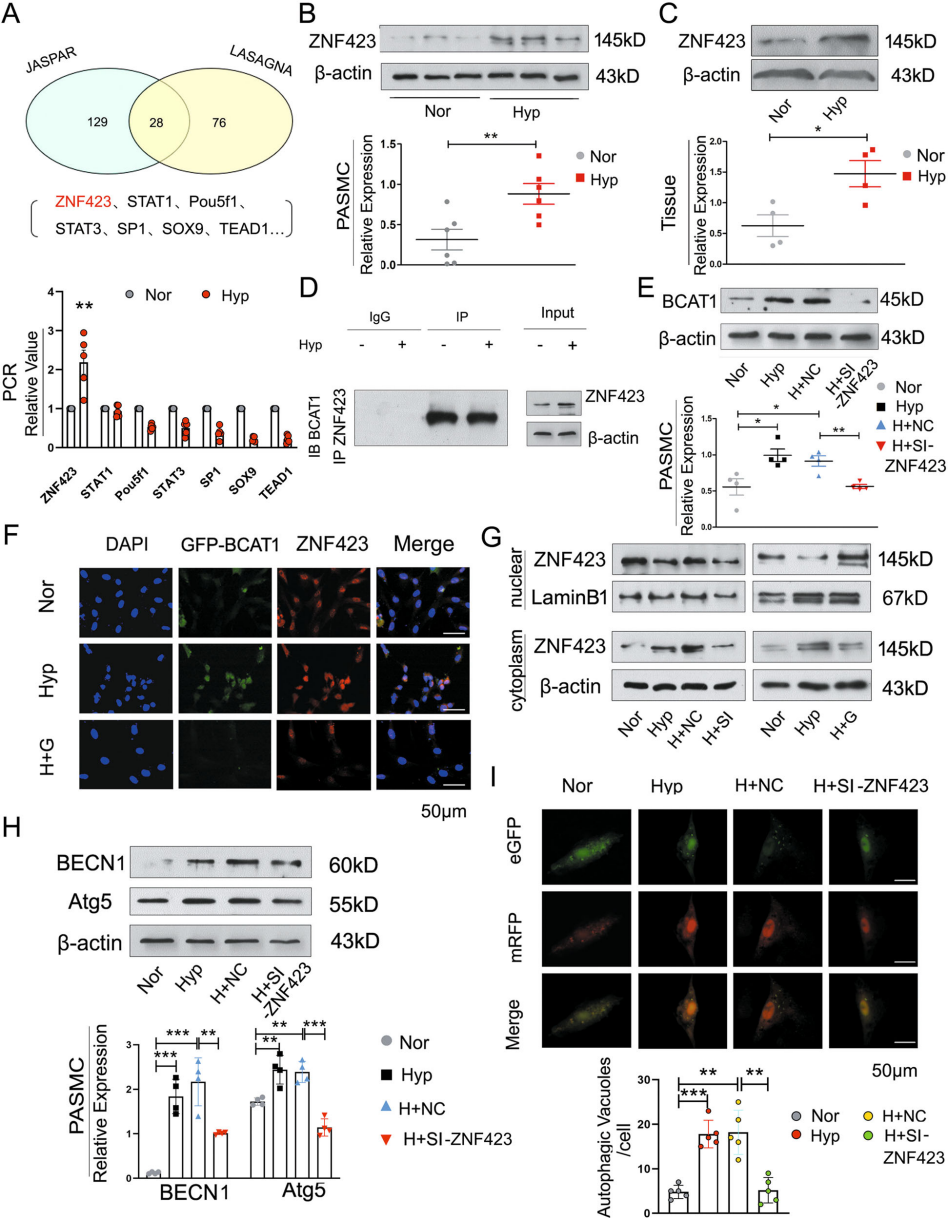
Hypoxia leads to the transfer of ZNF423 from the nucleus to the cytoplasm, where it bound BCAT1 to promote autophagy activity. **a** Bioinformatics analysis of proteins associated with BCAT1. Upside: According to the JASPAR database and LASAGNA-Search 2.0 database, there was 28 genes that may bind to bcat1, and the binding ability of ZNF423, STAT1, Pou5f1, STAT3, SP1, SOX9, and TEAD1 was strong. Underside: RT-PCR analysis of the mRNA levels of ZNF423, STAT1, Pou5f1, STAT3, SP1, SOX9, and TEAD1 with rat β-actin serving as the standard in PASMCs under NOR or HYP for 24 h (*n* = 5). **b** Western blot analysis of the expression of ZNF423 in PASMCs under NOR or HYP for 24 h (*n* = 6). **c** ZNF423 protein levels were assayed in pulmonary arterial tissues of hypoxic model rats (*n* = 4). **d** Coimmunoprecipitation of whole-cell lysates of PASMCs exposed to normoxia or hypoxia for 24 h with anti-ZNF423, followed by probing with anti-BCAT1 (*n* = 3). **e** Western blot analysis of BCAT1 expression in PASMCs transfected with ZNF423 siRNA under NOR or HYP for 24 h (*n* = 4). **f** PASMCs were exposed to HYP for 24 h, and the colocalization between BCAT1 and ZNF423 was determined by immunofluorescence. GFP-BCAT1 (green), ZNF423 (red), and DAPI (blue). Scale bar = 50 μm (*n* = 3). **g** The translocation of ZNF423 between the nucleus and cytoplasm in PASMCs transfected with BCAT1 siRNA or gabapentin (*n* = 3). **h** Western blot analysis of the expression of BECN1 and Atg5 in PASMCs transfected with ZNF423 siRNA under HYP for 24 h (*n* = 4). **i** Autophagic flux of PASMCs cotransfected with eGFP-mRFP-LC3 plasmid and control siRNA or ZNF423 siRNA under HYP for 24 h. Scale bar = 50 μm (*n* = 5). Nor normoxia, Hyp hypoxia, H + G hypoxia plus gabapentin, H + NC hypoxia plus control siRNA, H + SI hypoxia plus BCAT1 siRNA, H + si-ZNF423 hypoxia plus ZNF423 siRNA, IP immunoprecipitation, IB immunoblotting. Statistical analysis was performed with one-way ANOVA or the Student’s *t* test. All values are presented as the mean ± SEM. *p < 0.05; **p < 0.01; ***p < 0.001.

We further investigated the molecular mechanism by which hypoxia regulates BCAT1 expression in PASMCs. First, we predicted 28 kinds of proteins that might bind to BCAT1 through two bioinformatics databases, and then we selected seven genes with strong binding ability for further experimental analysis. The expression of zinc finger protein 423 (ZNF423) in hypoxic PASMCs was significantly enhanced by RT-PCR (Fig. 5a). Next, we examined the expression of ZNF423 in hypoxic PASMCs and the pulmonary arterial tissues of hypoxic model rats by Western blotting and found that ZNF423 expression was elevated in both cells and tissues compared with that in the control group (Fig. 5b, d). By coimmunoprecipitation assay, the BCAT1 protein was found to be highly enriched in ZNF423-precipitated samples compared with that in the control group, which confirmed the binding of BCAT1 and ZNF423 (Fig. 5d). After PASMCs were transfected with ZNF423 siRNA and cultured under hypoxia, we found that the expression of BCAT1 was decreased, suggesting that ZNF423 is an upstream protein of BCAT1 (Figs. 5e and S5d). BCAT1 and ZNF423 colocalization was tested by immunofluorescence experiments. Again, the results clearly indicated that BCAT1 colocalized with ZNF423 in hypoxic PASMCs (Fig. 5f).

Next, BCAT1 is expressed mainly in the cytoplasm^39^. We examined the expression location of ZNF423 by nuclear and cytoplasmic protein extraction experiments and found that ZNF423 was transferred from the nucleus to the cytoplasm in hypoxic PASMCs. After the inhibition of BCAT1 with a BCAT1 inhibitor or siRNA-mediated interference, we found that the inhibition or interference of BCAT1 could reverse the transfer of ZNF423 from the nucleus in hypoxic PASMCs, but the same result was not observed in the control (Fig. 5g). The above results showed that ZNF423 was transferred from the nucleus to the cytoplasm, where it bound BCAT1. The expression of autophagy-related proteins was reduced when only ZNF423 siRNA was used to treat PASMCs under hypoxic conditions (Fig. 5h). PASMCs were cotransfected with eGFP-mRFP-LC3 plasmid and ZNF423 siRNA and treated with hypoxia, following which we found that autophagosomes were significantly reduced after the interference of ZNF423 compared with those in the control group (Fig. 5i). Based on the above data, we confirmed that ZNF423 entered the cytoplasm of hypoxia-induced PASMCs, where it bound BCAT1 and promoted BCAT1 expression to induce the activation of autophagy.

### ZNF423 maintains the stable expression of BCAT1 in hypoxic PASMCs by binding the AREs of the BCAT1 mRNA 3′-UTR

**Fig. 6:**
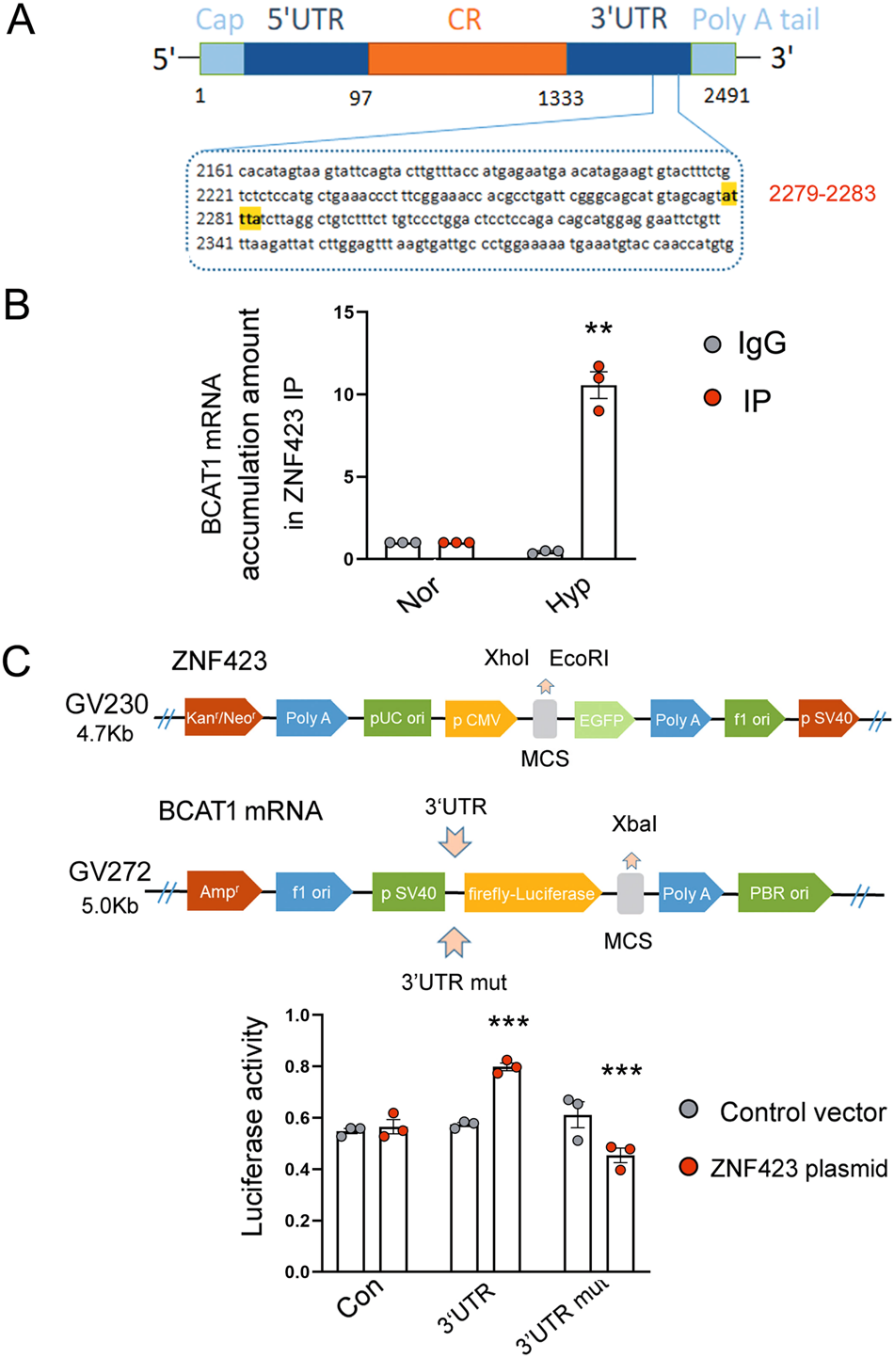
ZNF423 maintaines the stable expression of BCAT1 by binding the AU-rich elements (AREs) of the 3′-UTR of BCAT1 mRNA in hypoxic PASMCs. **a** The binding sites for ZNF423 in the 3′-UTR of BCAT1 mRNA. **b** The correlation between ZNF423 and BCAT1 mRNA was detected by real-time PCR after RNA immunoprecipitation (RIP) (*n* = 3). **c** Reporter constructs containing luciferase, and the 3′-UTR of BCAT1 mRNA and mutated 3ʹ-UTR of BCAT1 mRNA were used to estimate the activity of various luciferase reporter genes (*n* = 3). Nor normoxia, Hyp hypoxia, Con con083 control vector, 3′-UTR 3′-UTR luciferase reporter plasmid, 3′-UTR mut 3′-UTR ARE mutant luciferase reporter plasmid. Statistical analysis was performed with two-way ANOVA. All values are presented as the mean ± SEM. **p < 0.01; ***p < 0.001.

Finally, we aimed to explore the mechanism by which ZNF423 increases BCAT1 expression. Previous reports have indicated that zinc finger proteins are not only DNA-binding transcription factors but also RBPs that can bind AREs in 3′-UTRs to participate in the posttranscriptional regulation of proteins^40^. Using computational methods, for the first time, we identified the ARE region in the BCAT1 mRNA sequence (Fig. 6a). A typical type II sequence enriched in AUUUA was found in nucleotides 2279–2283 of the BCAT1 mRNA 3ʹ-UTR. By RNA immunoprecipitation (RIP) assay, we found that the BCAT1 PCR product was highly enriched in the ZNF423-precipitated sample but not the control IgG sample (Fig. 6b). To further confirm the binding of BCAT1 mRNA to ZNF423, we established a BCAT1 mRNA 3′-UTR luciferase reporter plasmid. Overexpression of ZNF423 in hypoxic PASMCs transfected with BCAT1 mRNA 3′-UTR fluorescent plasmid significantly increased the fluorescence activity compared with that of the control group transfected with the blank plasmid (Fig. 6c). More importantly, we also established a luciferase reporter plasmid to knock out AREs in the BCAT1 mRNA 3′-UTR. After the knockout of AREs via transfection of the 3′-UTR mutant plasmid, the upregulated fluorescence activity of ZNF423 was eliminated (Fig. 6c). Taken together, these findings indicated that ZNF423 could promote BCAT1 mRNA stability by binding AREs at the BCAT1 mRNA 3′-UTR (nucleotides 2279–2283) in hypoxic PASMCs.

## Discussion

In this study, using both in vitro and in vivo approaches, we have highlighted three new concepts. First, we discovered that hypoxia upregulates BCAT1 expression in PASMCs. Second, BCAT1-induced PASMC autophagy under hypoxic conditions was found to occur through the binding of BCAT1 to the ERs pathway protein IRE1, leading to activation of the downstream XBP1 and RIDD pathways. Third, we proved for the first time that the ZNF423 protein is located upstream of BCAT1 and regulates BCAT1 expansion via binding AREs of the BCAT1 mRNA 3′-UTR. Our results provide substantial evidence of the role and mechanism of BCAT1 in hypoxia-induced PASMC autophagy.

The activation of autophagy, which is involved in the proliferation and migration of PASMCs induced by hypoxia, was alleviated by treatment with the autophagy inhibitor 3-mA^41^. In addition, ATG5 siRNA could inhibit the proliferation of PASMCs by directly blocking autophagy^42^. Although a few studies have reported data on the role of autophagy in PASMCs, the specific molecular regulatory mechanism is not completely understood. Notably, BCAT1 plays an important role in inhibiting cell proliferation and promoting cell apoptosis in mammalian cells^43^. Other reports have identified BCAT1 as a target gene of c-Myc, which induces cell proliferation, migration and invasion in nasopharyngeal carcinoma^19^. However, that the involvement of BCAT1 in the process of autophagy has not been reported. Hence, we determined that BCAT1 is a key driver of autophagy in PASMCs via both in vivo and in vitro experiments.

ER stress is a protective cellular stress response that occurs when unfolded proteins accumulate in the ER lumen. Recently, it was reported that the ERs pathway is activated in many known pulmonary arterial processes, including hypoxia, viral infection, BMPRII mutation, inflammation, and Notch induction^44–49^. Meanwhile, different models of hypoxia have shown that ERs activates autophagy to clear damaged proteins and to reduce the stress response^50–52^. However, the regulation of autophagy by the ERs signaling pathway in PASMCs has not been studied. To further understand the signaling pathways that mediate this response, we investigated the role of the ERs signaling pathway in BCAT1-mediated autophagy in PASMCs. Indeed, our results proved that BCAT1 regulates autophagy activation in response to hypoxia via activating the ERs signaling pathway.

ER stress activates the UPR, which can be divided into three branches, the PERK, IRE-1, and ATF6 branches^50^. Our results indicated that an abnormal increase in BCAT1 expression favors ERs pathway protein and autophagy activation. Therefore, identifying the ERs pathway that is a key regulator of BCAT1-induced autophagy in PASMCs is an urgent problem that needs to be solved. Our data showed binding between BCAT1 and IRE1. IRE1 is a D-bifunctional protein consisting of one kinase domain and an RNase domain involved in two downstream signaling pathways, XBP1 splicing and the RIDD pathway^53–55^. Following the inhibition of BCAT1 with BCAT1 siRNA or the BCAT1 inhibitor gabapentin, the expression of XBP1-s, sparc, pmp2, and scara3 was significantly inhibited (Fig. 4c, d). These results confirmed that the expression of BCAT1 was elevated in hypoxic PASMCs and that BCAT1 bound IRE1 on the ER to activate the ERs pathway, following which autophagy was activated by the XBP1 and RIDD pathways. To the best of our knowledge, this is the first report to identify the ERs signaling pathway involved in PASMC autophagy.

Zinc finger proteins are mostly regarded as DNA-binding transcription factors that play a role in DNA binding^56,57^. Zinc finger proteins can also act as RBPs and regulate RNA metabolism^40^. More importantly, RBPs can bind AREs, which are important sites for posttranscriptional regulation, in the 3′-UTRs of mRNA. The binding of AREs and RBPs stabilizes the transcript, thereby increasing protein translation^58,59^. Interestingly, we originally identified ZNF423, a zinc finger protein, as an RBP. By bioinformatics analysis, we found that the 3′-UTR of BCAT1 mRNA contains type II AREs (one or several AUUUA pentamers in the 3′-UTR)^60^. Through a series of experiments, we verified that ZNF423, an RBP, bound AREs within nucleotides 2279–2283 of the 3′-UTR of BCAT1 mRNA, which promoted the translation and high-level expression of BCAT1 in hypoxic PASMCs. We also found that hypoxia caused ZNF423 to shuttle between the cytoplasm and nucleus, which effectively revealed the key reason for BCAT1 accumulation in the cytoplasm under hypoxia. It has been reported that RBPs can be exported from nuclei into the cytoplasm^61^. These results confirmed the feasibility of our experimental results.

Our study still has some limitations. The BCAT1 enzyme initiates the catabolism of BCAAs. For example, BCAT1, a critical enzyme for αKG homeostasis, links BCAA metabolism in human acute myeloid leukemia^62^. Therefore, whether the metabolites of BCAT1 are also involved in the regulation of autophagy induced by hypoxia remains unknown. We will further strengthen our conclusions through the exogenous addition of metabolites in subsequent experiments.

**Fig. 7:**
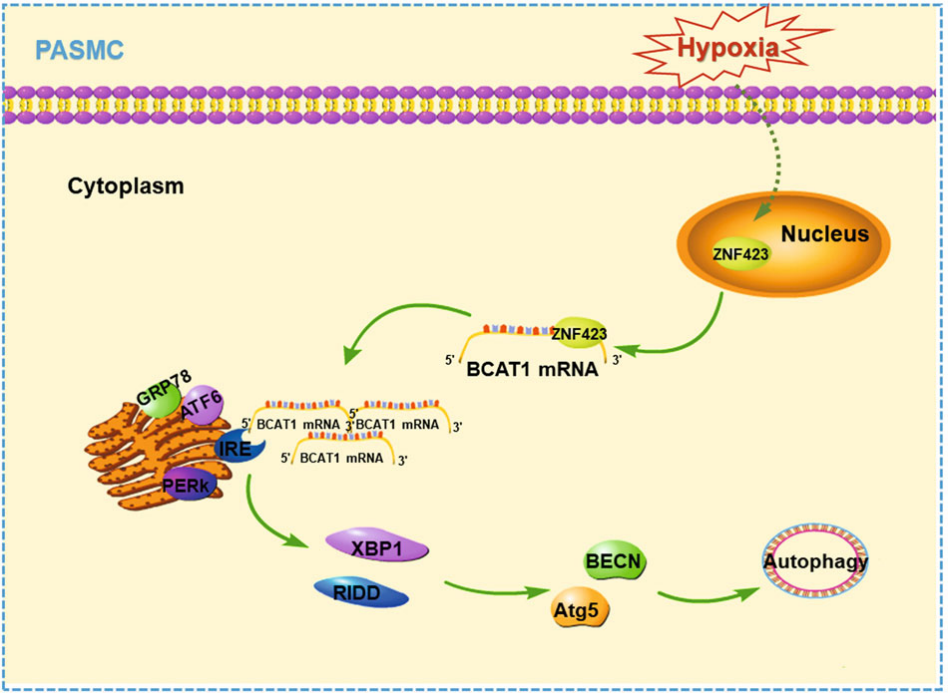
The proposed mechanism for the role of BCAT1 in PASMC autophagy. **a** Hypoxia causes ZNF423 to shuttle from the nucleus to the cytoplasm, where it binds the AREs of the 3′-UTR of BCAT1 mRNA, promoting BCAT1 translation and increasing BCAT1 expression. Then, BCAT1 binds IRE1 on the endoplasmic reticulum to activate RE stress pathway proteins and the XBP1 and RIDD pathways downstream of IRE1, constituting an IRE1-XBP1-RIDD cell axis, ultimately inducing autophagy activation in PASMCs.

To conclude, we have identified the key role of BCAT1 in the regulation of autophagy in hypoxic PASMCs for the first time. Simultaneously, we have revealed the key molecular mechanisms of BCAT1 and ZNF423 by which autophagy is activated through the IRE1 pathway in hypoxic PASMCs (Fig. 7). Our results provide novel clues for the study of the molecular regulatory mechanism and signaling pathway of autophagy in PASMCs and may provide a novel and important basis for the diagnosis and clinical treatment of lung diseases.

## Materials and methods

### Animals and lung tissue preparation

All animal experiments were conducted in accordance with the NIH Guidelines for the Care and Use of Laboratory Animals and approved by the Institutional Animal Care and Use Committee (approval number by Animal Committee: HMUDQ20200601). Adult male wistar rats with a mean weight of 130 g were obtained from the Harbin Medical University Experimental Animal Center. Rats were randomly allocated into normoxia, monocrotalin (MCT) and hypoxia groups. In the MCT groups, rats were injected subcutaneously 40 mg/kg MCT in a single time for 21 days. In hypoxia groups administered fractional inspired oxygen (Fi,O_2_) 0.21 and 0.12 for 21 days. The rats were intraperitoneally injected with gabapentin (1 mg/kg), twice a week for 3 weeks. After the exposure period, pulmonary arteries were extracted, and lung tissues were taken for immunofluorescent treatment and western blot analysis.

### Cell isolation and culture

The primary pulmonary arteries of adult Wistar rats (100–150 g) were extracted. The chopped tissues were digested for two hours in 2 mg/ml collagenase II (Sigma, c6885). PASMCs were cultured in culture medium containing 20% fetal bovine serum^63^ and 1% penicillin and streptomycin in a 5% CO_2_ humidified incubator at 37 °C. Before each experiment, the cells were incubated in DMEM 5% serum for 24 h. Cells in hypoxic culture were incubated in a Tri-Gas Incubator (Heal Force, Shanghai, China) with a gas mixture containing 92% N2–5% CO_2_–3% O_2_ for 24 h.

### Antibodies and reagents

The following antibodies and reagents were used: anti-BCAT1 (Boster, BA2190), anti-α-SMA (Boster, BM0002), anti-BCAT2 (Boster, BA3633), anti-BECN1 (Boster, PB0014), gabapentin (Selleck, S2133), anti-ATG5 (Boster, BA3525-2), anti-LC3B (Abcam, ab48394), anti-PERK (Boster, A01992), anti-IRE-1 (Boster, A00683-1), anti-ATF6 (Boster, BM0002), anti-GRP78 (Santa Cruz Biotechnology, sc-376768), anti-XBP1 (Boster, PB0487), anti-ZNF423 (Abcam, ab169096), ER-Tracker Red (Beyotime, C1041), and 4-PBA (Santa Cruz Biotechnology, sc-200652).

### siRNA construction and transfection or chemical treatment of PASMCs with siRNA and inhibitor

PASMCs were transfected with siRNAs designed and synthesized by GenePharma (China) to knock out the target protein. Nontargeted control siRNA was used as a negative control (NC).

siRNAs with following sequences were used:

si-BCAT1: 5′-GCGAGAGACACAUCACCAUTT-3′, 3′-AUGGUGAUGUGUCUCUCGCTT-5′; si-IRE1: 5′-UCCAUCAAGUGGACUUUAATT-3′, 3′-UUAAAGUCCACUUGAUGGATT-5′; si-XBP1: 5′-CAAGCUGGAAGCCAUUAAUTT-3′, 3′-AUUAAUGGCUUCCAGCUUGTT-5′; si-ZNF423: 5′-GCCGCGAUCGGUGAAAGUUTT-3′, 3′-AACUUUCACCGAUCGCGGCTT-5′; and si-NC: 5′-UUCUUCGAACGUGUCACGUTT-3′, 3′-TTAAGAGGCUUGCACAGUGCA-5′. The transfection methods used have been previously described^64^. PASMCs were transfected with siRNA or treated with gabapentin inhibitor (20 mM), 4-PBA (20 mM) and Bafilomycin A1(Baf-A1) (100 nM, Selleck, S1413). The cells were transfected with siRNA using X-tremeGENE siRNA transfection reagent (Roche Applied) according to the manufacturer’s protocol. In summary, 3.75 μL of siRNA and 5 μL of X-tremeGENE siRNA transfection reagent were separately diluted in 100 μL of serum-free Opti-MEM-1 for 5 min and then mixed and incubated for 15 min at room temperature. After incubation with the cells for 4–6 h, the medium was replaced with medium containing 5% fetal bovine serum, and the cells were used after hypoxia treatment.

### Plasmid construction and transfection

The BCAT1 plasmid (NM_017253), ZNF423 plasmid (NM_053583), BCAT1 3′-UTR luciferase reporter plasmid (NM_017253-3utr), BCAT1 3′-UTR ARE region mutant luciferase reporter plasmid (NM_017253-3utr-mut), and control plasmid were ordered from Shanghai GeneChem (China). The constructed plasmid was verified by sequencing. Plasmids were transfected with Lipofectamine^®^ 2000 Reagent (Life technologies, 11668) according to the instructions.

### Western blot analysis

The pulmonary arterial tissues of hypoxia model rats were homogenized in a handheld microtissue grinder with lysis buffer (50 mM Tris, pH 7.4, 150 mM NaCl, 1% Triton X-100, 1 mM EDTA, and 2 mM PMSF) on ice. The homogenates were centrifuged at 16,099×*g* for 15 min at 4 °C. Then, the supernatant was pumped into a centrifuge tube and stored at −80 °C for subsequent experiments. PASMC proteins were solubilized and extracted with lysis buffer (50 mM Tris, pH 7.4, 150 mM NaCl, 1% Triton X-100, 1 mM EDTA, and 2 mM PMSF) and then incubated for 30 min on ice. The lysates were sonicated and centrifuged at 16,099×*g* for 15 min, and the insoluble fractions were discarded. The soluble fractions were saved as described previously. Protein samples were electrophoresed on an sodium dodecyl sulfate polyacrylamide gel and transferred to nitrocellulose membranes. The membranes were blocked in blocking buffer (20 mM Tris, pH 7.6, 150 mM NaCl and 0.1% Tween 20) containing 5% nonfat dry milk. The membranes were overnight at 4 °C with primary antibodies and reacted with the appropriate horseradish peroxidase-conjugated secondary antibodies. Then, Super ECL reagent (HaiGene, M2301) was used for protein detection.

### Immunofluorescence staining

PASMCs were cultured under normoxia or hypoxia for 24 h after transfection or drug administration, and the lung tissues of hypoxia model rats after treatment were embedded in paraffin. The cells or tissue sections were immobilized in 4% paraformaldehyde, permeabilized by treatment with 0.01% Triton X-100 and blocked with 3% normal bovine serum. The cells or tissue sections were then incubated overnight at 4 °C with anti-BCAT1 (1:100), anti-α -SMA (1:100), anti-BECN (1:100), and anti-ATG5 (1:100). The PASMCs were then incubated with the appropriate FITC-conjugated secondary antibody, Cy3-conjugated secondary antibody, and DAPI. Images were captured with a confocal laser scanning microscope.

### Autophagic flux monitoring assay

mRFP-GFP-LC3 adenovirus obtained from Hanbio Biotechnology (HB-AP210 0001) was applied to PASMCs treated with siRNA under hypoxia for 24 h to monitor cell autophagic flux. The experimental procedure was carried out according to the manufacturer’s instructions.

### ER-tracker red staining

ER-Tracker Red, a red fluorescent probe of the ER with membrane permeability that can be used to specifically stain the ER in living cells, was purchased from Beyotime (c1041). Briefly, the cells were washed with 1× PBS after hypoxic treatment and plated. One microliter of ER-Tracker Red was added to 1 ml of dilute ER-Tracker Red and mixed to make an ER-Tracker Red working solution. The ER-Tracker Red working solution required preincubation at 37 °C before use. The washing solution was removed, and the prepared working solution was added and incubated with the cells at 37 °C for 15–30 min. The ER-Tracker Red staining solution was removed, and the cells were washed 1–2 times with cell culture. This was followed by observation with a fluorescence microscope or laser confocal microscope.

### Nuclear and cytoplasmic protein extraction assay

A nuclear and cytoplasmic protein extraction kit was purchased from Beyotime (P0028). The experiment was performed according to the manufacturer’s instructions.

### Coimmunoprecipitation

After the cell model was successfully established, the cells were first washed three times with precooled PBS, and 1 ml of cell lysate plus PMSF (1:100) was then added. The cells were scraped with a precooled scraper, and the cell suspension was collected in a 1.5 ml EP tube that was placed in ice on shaking platform and incubated with slow shaking for 30 min. The cell suspension was then subjected to high-speed centrifugation at 4 °C and 15,000 rpm/min for 30 min. The supernatant was transferred to a new EP tube, and 100 µl was taken and used as a blank control group. The remaining 50 µl was combined with protein A + G agarose (Beyotime, p2012) and shaken slowly for 4 h at 4 °C, following which the mixture was centrifuged at 4 °C and 15,000 rpm for 15 min. The supernatant was transferred to a new tube, and 100 µl was taken and used as the IgG control group. One nanogram of the antibody of interest was added to the remaining supernatant, incubated at 4 °C for 6 h, and shaken overnight after the addition of 50 µl of protein A + G agarose in a 4 °C shaker. The next day, the samples underwent high-speed centrifugation at 4 °C and 5000 rpm for 5 min. The supernatant was discarded, and the pellet was then washed 4 times with 1 ml of precooled PBS and spun between each wash with a high-speed centrifuge at 4 °C and 5000 rpm. Finally, the supernatant was discarded, and an equal amount of 2× loading buffer (10 µd/ng) was added. The agarose–antigen–antibody complex was mixed, heated at 100 °C for 5 min, and detected by Western blotting.

### RIP analysis

Binding between mRNA and protein was analyzed using an Imprint^®^ RNA immunoprecipitation kit (Sigma, RIP-12RXN) according to the manufacturer’s instructions.

### Dual-luciferase reporter gene assay

A dual-luciferase reporter gene assay kit was purchased from Beyotime (RG027). Briefly, after successful cell modeling, the reporter cell lysate was fully lysed and centrifuged at 10,000–15,000 × *g* for 5 min, and the supernatant was aspirated for detection. An appropriate amount of Renilla luciferase assay buffer was added, and the Renilla luciferase assay substrate (100×) was added at 1:100 to prepare a Renilla luciferase assay working solution. One-hundred microliters was removed from each sample, 100 µl of firefly luciferase detection reagent was added, and a multifunction microplate reader was used to measure the relative light units (RLUs). The reporter cell lysate was used as a blank control. One-hundred microliters of Renilla luciferase was added and mixed, flowing which the RLUs were measured to test the working solution.

### Histological and morphometric analyses

The lung tissues of rats were fixed in 4% paraformaldehyde for 24 h. Then fixed tissues were dehydrated, cleared, and embedded in paraffin base. The Paraffin tissue is cut into 5 μm. These sections were stained with hematoxylin and eosin (HE) or Masson’s trichrome stain. In immunohistochemistry assay, the sections tissue were dewaxed, restored and incubated with antibodies overnight. Tissue sections were incubated with fluorescence secondary antibodies. Then, the tissue sections were stained with 3, 3-diaminobenzidine (DAB) and restrained with hematoxylin. Appearance of tissues were recorded by a fluorescence microscope (Nikon) equipped with a digital camera.

### Hemodynamic analysis and ventricular weight measurement

The rats were weighed and then anesthetized. The catheter was inserted into the right ventricle, RV pressures couble be recorded by computer to evaluate the level of right ventricular hypertrophy. The heart was cut into the RV free wall and the left ventricle (LV) plus septum, then weighted separately. The level of right ventricular hypertrophy was determined with the ratio RV/LV + septum.

### Microfil perfusion

The lung tissues were fixed with formalin and infused with microfil (MV-122, Flow Tech, Inc. MA, USA). Alcohol-methyl salicylate clearing was implemented, that made the lung tissues transparent according to the manufacturer’s instructions.

### Echocardiography

Small animal echocardiography was performed with a Vevo2100 imaging system (Visual Sonics Inc., Toronto, ON, Canada) and a 30 MHz probe. The Rats were anesthetized with 4% chloral hydrate. Then, obtained stable information from instrument record results, such as the pulmonary arterial velocity time integral, pulmonary arterial pre-ejection time, and pulmonary arterial ejection time. All measurements were in line with the American Society of Echocardiography guidelines.

### Statistical analysis

Statistical analyses were performed using GraphPad Prism 8 (GraphPad Software, LaJolla, CA, USA) software. The relevant data are presented as the means ± SEM. Statistical analysis was performed with Student’s *t* test or one-way ANOVA followed by Tukey’s test where appropriate. Differences for which *p* < 0.05 were significant. All experiments were performed in triplicate.

## Supplementary information

Supplementary Figure Legends

Supplementary Figure S1

Supplementary Figure S2

Supplementary Figure S3

Supplementary Figure S4

Supplementary Figure S5

Table 1-Reagent

Table 2-JASPAR data

Table 3-LASAGNA data
