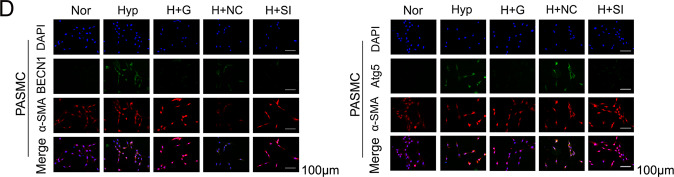# Correction: BCAT1 binds the RNA-binding protein ZNF423 to activate autophagy via the IRE1-XBP-1-RIDD axis in hypoxic PASMCs

**DOI:** 10.1038/s41419-021-04466-1

**Published:** 2021-12-23

**Authors:** Wei Xin, Min Zhang, Yang Yu, Songlin Li, Cui Ma, Junting Zhang, Yuan Jiang, Yiying Li, Xiaodong Zheng, Lixin Zhang, Xijuan Zhao, Xuzhong Pei, Daling Zhu

**Affiliations:** 1 College of Pharmacy, Harbin Medical University, Harbin, 150081 P.R. China; 2 Central Laboratory of Harbin Medical University (Daqing), Daqing, 163319 P.R. China; 3 Division of Cardiology and Hubei Key Laboratory of Genetics and Molecular Mechanisms of Cardiological Disorders, Tongji Hospital, Tongji Medical College, Huazhong University of Science and Technology, Wuhan, 430030 P.R. China; 4 College of Pharmacy, Harbin University of Commerce, Harbin, 150076 P.R. China; 5 College of Medical Laboratory Science and Technology, Harbin Medical University (Daqing), Daqing, 163319 P.R. China; 6 Department of Genetic and Cell Biology, Harbin Medical University (Daqing), Daqing, 163319 P.R. China; 7 State Province Key Laboratories of Biomedicine-Pharmaceutics of China, Daqing, 163319 P.R. China; 8 Key Laboratory of Cardiovascular Medicine Research, Ministry of Education, Harbin Medical University, Harbin, 150081 P.R. China

**Keywords:** Autophagy, Post-translational modifications

Correction to: *Cell Death and Disease* 10.1038/s41419-020-02930-y, published online 16 September 2020

The original version of this article unfortunately contained a mistake in figure 2d. The correct figure can be found below. The authors apologize for the mistake. The original article has been corrected.