# Supplementary material for: BCAT1 binds the RNA-binding protein ZNF423 to activate autophagy via the IRE1-XBP-1-RIDD axis in hypoxic PASMCs
Source: Cell Death Dis. 2020 Sep 16;11(9):764. doi: 10.1038/s41419-020-02930-y (PMC7494854; doi:10.1038/s41419-020-02930-y)
Supplement: Supplementary file 1 — Supplementary Figure Legends [file 41419_2020_2930_MOESM1_ESM.docx]

**Figure S1. Gabapentin specifically inhibited hypoxia-regulated BCAT1 expression in vivo. a** Modeling diagram of two kinds of hypoxia rats. **b.c** HE staining and masson stain detected for the morphological changes of pulmonary vessels (n=3). Scale bars: 100μm. **d** Gabapentin treatment could reverse the vascular thickening caused by hypoxia (n=8). **e** Gabapentin treatment reduced pulmonary vascular density (n=8). **f** Gabapentin treatment depressed mean RVSP (n=8). **g** Gabapentin treatment reducesd the ratio of RV weight (RV/LV+S) (n=8). **h** Gabapentin treatment increased PAVTI (n=8). Nor, normoxia; Hyp, hypoxia; Mct, monocrotalin; Hyp+Gap, hypoxia plus gabapentin; Mct+Gap, monocrotalin plus gabapentin. Statistical analysis was performed with one-way ANOVA. All values are presented as mean±SEM. **p < 0.01, ***p < 0.001.

**Figure S2. There are no significant difference in BCAT2 expression in PASMCs and BCAT1 expression in PAECs.** **a** Western blot analysis of BCAT2 expression in PASMCs under hypoxia for 24 h (n=7). **b** Western blot analysis of BCAT1 expression in PAECs (n=6). Nor, normoxia; Hyp, hypoxia. Statistical analysis was performed with the Student's t-test. All values are presented as the mean±SEM. ns; not significant.

**Figure S3. Detection of autophagic flux and interference with XBP1 inhibits autophagic activity in hypoxic PASMCs.** **a** Western blot analysis of the expression of LC3B-II in PASMCs treated with bafilomycin A1 and gabapentin (n=3). **b** Western blot analysis of BECN1 and Atg5 expression in PASMCs transfected with XBP1 siRNA (n=5). Nor, normoxia; Hyp, hypoxia; H+Baf, hypoxia plus bafilomycin A1; H+Baf+G, hypoxia plus bafilomycin A1 plus gabapentin; H+NC, hypoxia plus control siRNA; H+Si-XBP1, hypoxia plus XBP1 siRNA; Statistical analysis was performed with one-way ANOVA. All values are presented as the mean±SEM. *p < 0.05; **p < 0.01; ***p < 0.001.

**Figure S4. BCAT1 don’t bind PERK, ATF6 or GRP78 in PASMCs. a.b and c** Coimmunoprecipitation of the whole-cell lysates of PASMCs exposed to normoxia or hypoxia for 24 h, with anti-PERK, anti-ATF6 and anti-GRP78, followed by probing with anti-BCAT1 (n=3). IP, immunoprecipitation; IB, immunoblotting.

**Figure S5. The interference and overexpression efficiency of several siRNAs and plasmid. a.b and c** Western blot analysis of the transfection efficiency of BCAT1, IRE1, XBP1 and ZNF423 in PASMCs (n = 4). **d** RT-PCR of the overexpression efficiency of BCAT1 (n = 6). Statistical analysis was performed with one-way ANOVA or the Student's t-test. All values are presented as the mean±SEM. *p < 0.05; **p < 0.01; ns, not significant.
