# Supplementary figures and images for: BCAT1 binds the RNA-binding protein ZNF423 to activate autophagy via the IRE1-XBP-1-RIDD axis in hypoxic PASMCs
Source: Cell Death Dis. 2020 Sep 16;11(9):764. doi: 10.1038/s41419-020-02930-y (PMC7494854; doi:10.1038/s41419-020-02930-y)

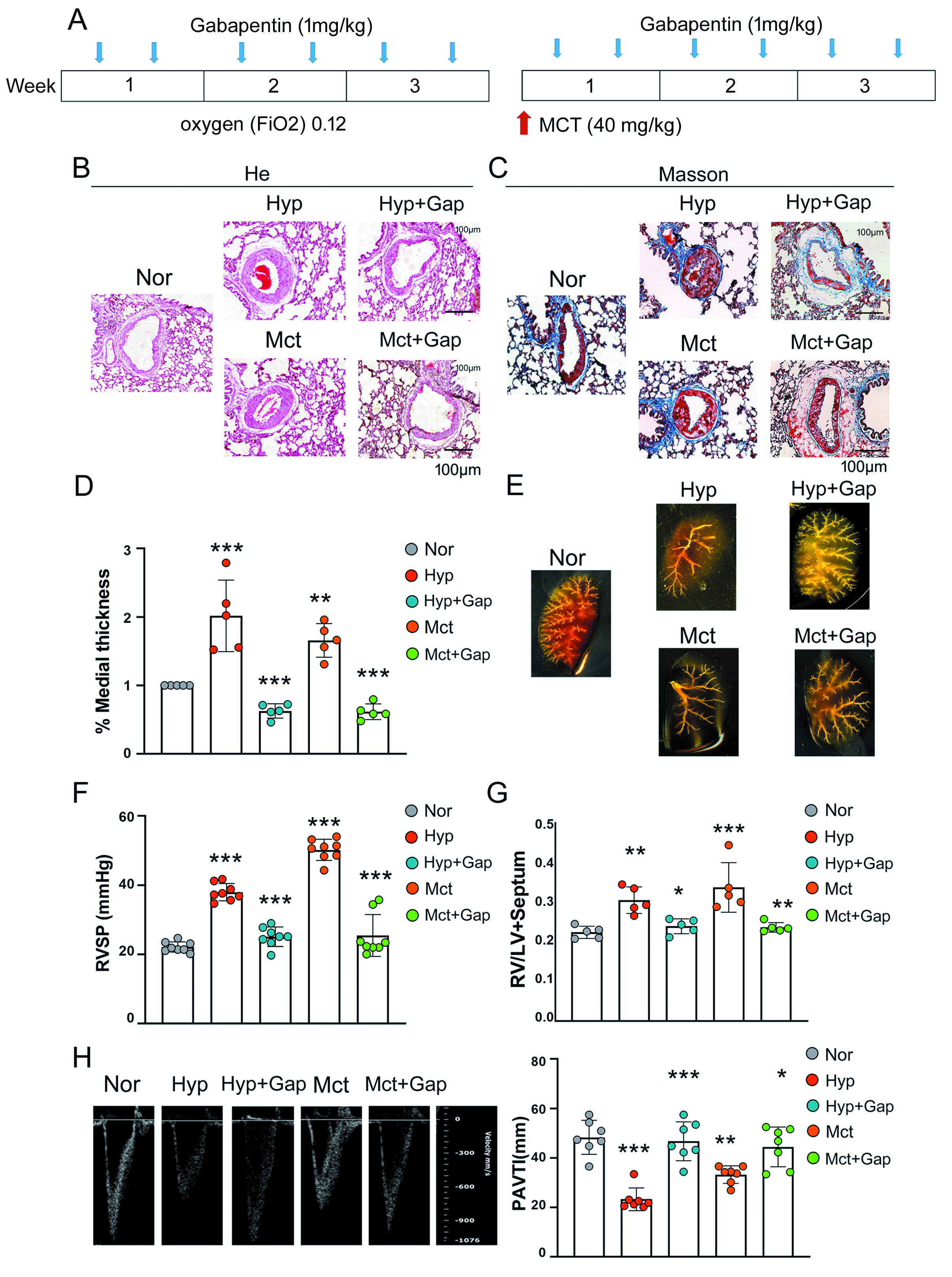

Supplement: Supplementary file 2 — Supplementary Figure S1 [file 41419_2020_2930_MOESM2_ESM.tif]

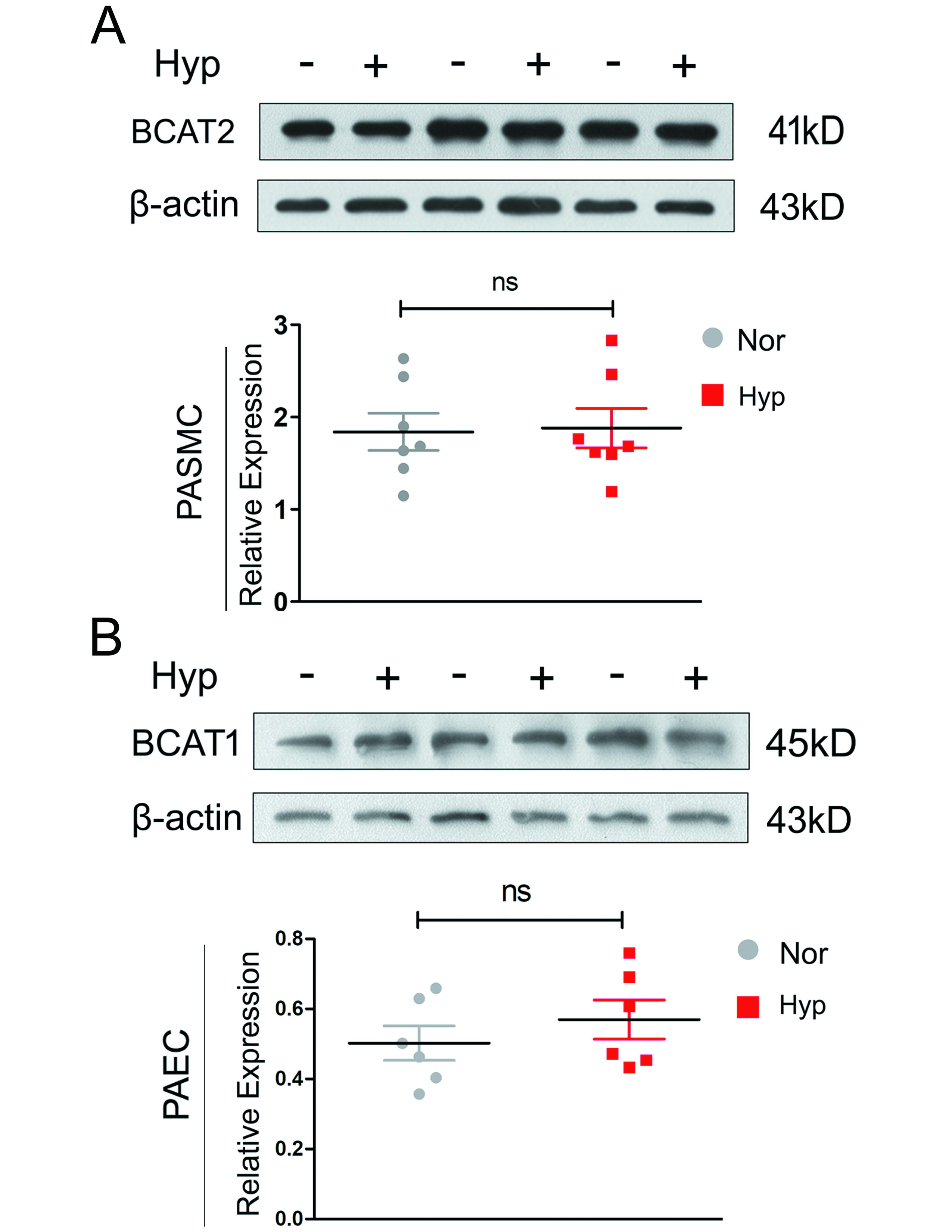

Supplement: Supplementary file 3 — Supplementary Figure S2 [file 41419_2020_2930_MOESM3_ESM.tif]

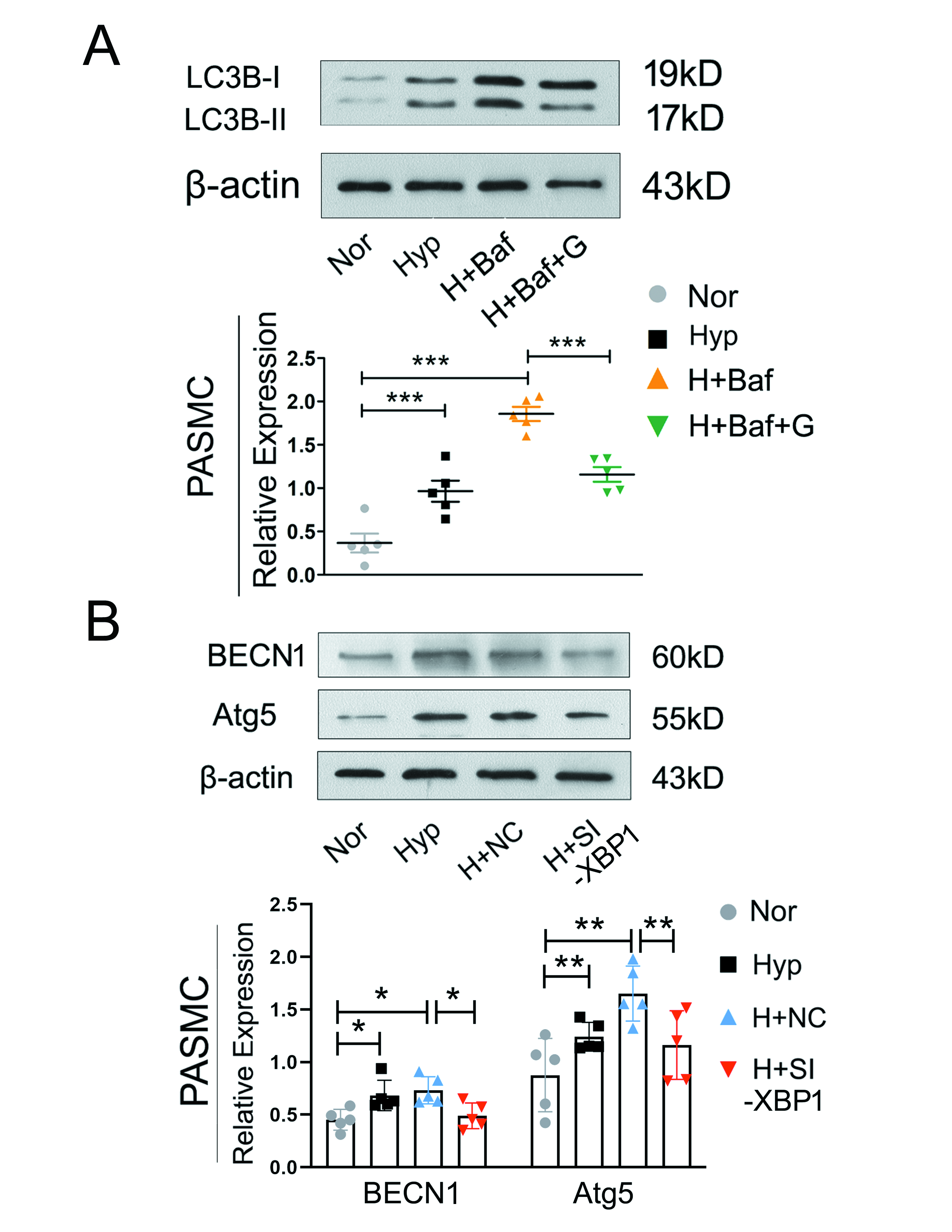

Supplement: Supplementary file 4 — Supplementary Figure S3 [file 41419_2020_2930_MOESM4_ESM.tif]

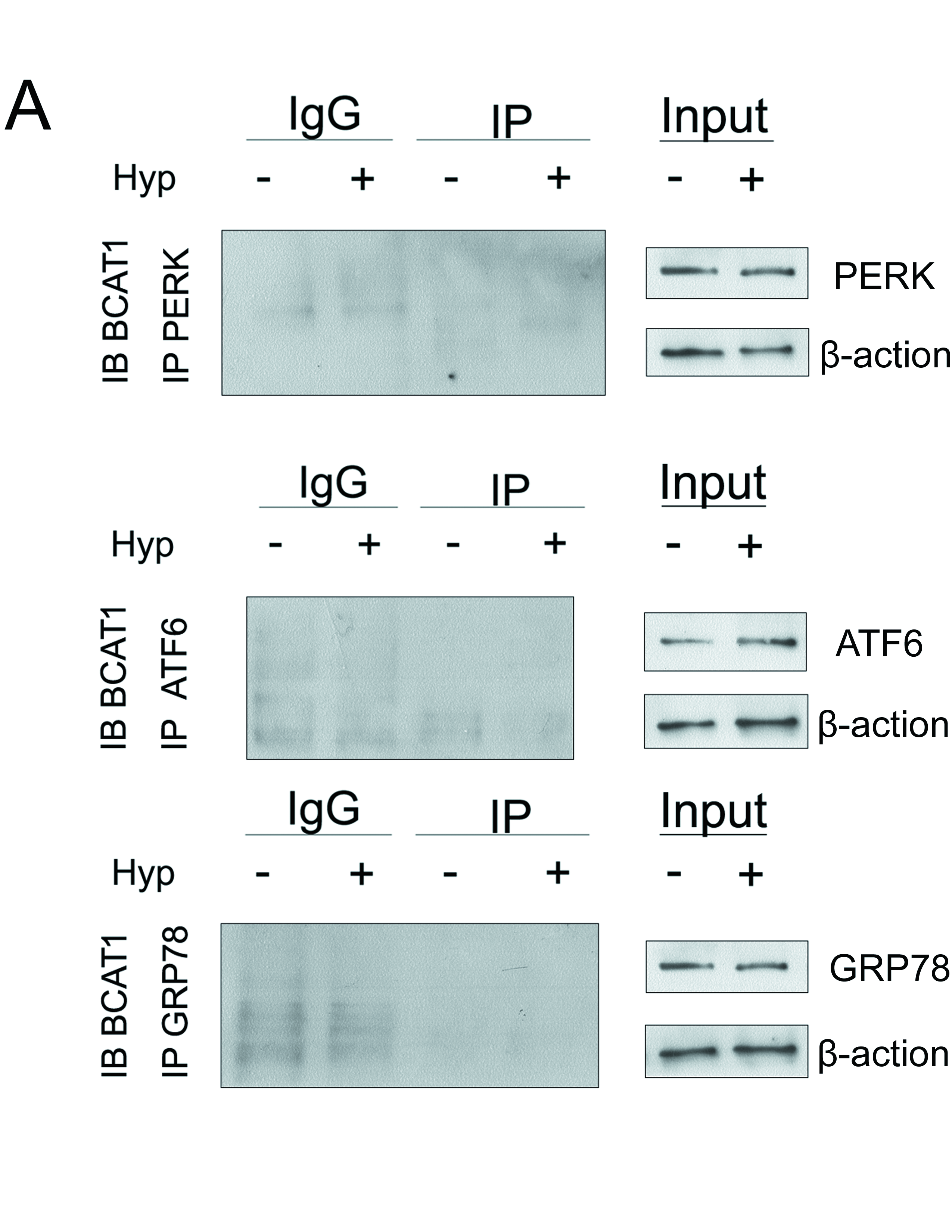

Supplement: Supplementary file 5 — Supplementary Figure S4 [file 41419_2020_2930_MOESM5_ESM.tif]

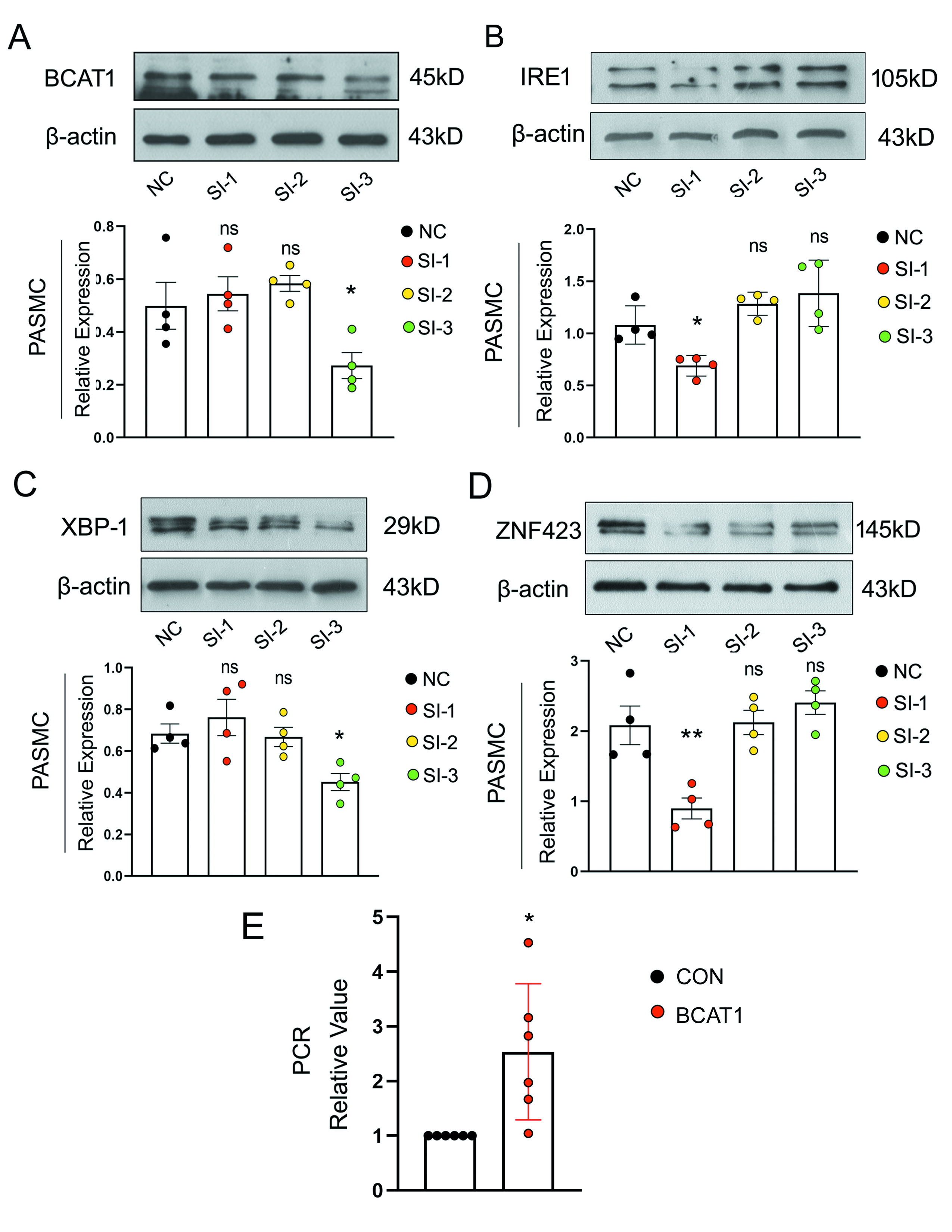

Supplement: Supplementary file 6 — Supplementary Figure S5 [file 41419_2020_2930_MOESM6_ESM.tif]
