# Supplementary material for: BCAT1 binds the RNA-binding protein ZNF423 to activate autophagy via the IRE1-XBP-1-RIDD axis in hypoxic PASMCs
Source: Cell Death Dis. 2020 Sep 16;11(9):764. doi: 10.1038/s41419-020-02930-y (PMC7494854; doi:10.1038/s41419-020-02930-y)
Supplement: Supplementary file 7 — Table 1-Reagent [file 41419_2020_2930_MOESM7_ESM.docx]

**Reagents and Tools Table**

| **Recombinant DNA** |  |  |
| --- | --- | --- |
| BCAT1 | GenePharma (China) | pEX-5 |
| NC-BCAT1 | GenePharma (China) | pEX-5 |
| ZNF423 (NM_053583) | GENECHEM | CMV-MCS-EGFP-SV40-Neomycin |
| Control -ZNF423 | GENECHEM | CMV-MCS-EGFP-SV40-Neomycin |
| BCAT1 (NM_017253-3utr) | GENECHEM | SV40-firefly_Luciferase-MCS |
| BCAT1 (NM_017253-3utr-mut) | GENECHEM | SV40-firefly_Luciferase-MCS |
| Control-BCAT1 | GENECHEM | SV40-firefly_Luciferase-MCS |
| **Antibodies** |  |  |
| Rabbit-anti-BCAT1 | Abcam | BA2190 |
| Mouse-anti-α-SMA | Boster | BM0002 |
| Rabbit-anti-BCAT2 | Boster | BA3633 |
| Rabbit-anti-BECN1 | Boster | PB0014 |
| Rabbit-anti-ATG5 | Boster | BA3525-2 |
| Rabbit-anti-LC3B | Abcam | ab48394 |
| Rabbit-anti-PERK | Boster | A01992 |
| Rabbit-anti-IRE-1 | Boster | A00683-1 |
| Rabbit-anti-ATF6 | Boster | BA2374 |
| Rabbit-anti-GRP78 | Santa Cruz Biotechnology | sc-376768 |
| Rabbit-anti-XBP1 | Boster | PB0487 |
| Rabbit-anti-ZNF423 | Abcam | ab169096 |
| **Oligonucleotides and other sequence-based reagents** |  |  |
| si-NC | GenePharma (China) | 5'-UUCUUCGAACGUGUCACGUTT-3' |
| si-BCAT1 | GenePharma (China) | 5'-GCGAGAGACACAUCACCAUTT-3' |
| si-IRE1 | GenePharma (China) | 5'-UCCAUCAAGUGGACUUUAATT-3' |
| si-XBP1 | GenePharma (China) | 5'-CAAGCUGGAAGCCAUUAAUTT-3' |
| si-ZNF423 | GenePharma (China) | 5'-GCCGCGAUCGGUGAAAGUUTT-3' |
| BCAT1(rat) | Nanjing jin si rui biotechnology co., LTD | sense 5’-TGCTGATGGTGGAGTGGA-3’, antisense 5’-CTGGCAGAGGTTGAGTAGGG-3’ |
| XBP1-s(rat) | Nanjing jin si rui biotechnology co., LTD | sense 5’-CTTCTCCCTTCAGCGACAT-3’, antisense 5’-TGGTGGGTGGCTTTAGAC-3’ |
| sparc(rat) | Nanjing jin si rui biotechnology co., LTD | sense 5’-CCCACCCACTTCCACTAC-3’, antisense 5’-CTTATGCAATTCCCGTTT-3’ |
| pmp2(rat) | Nanjing jin si rui biotechnology co., LTD | sense 5’-GTGGAATGTATAATGAAGGGTG-3’, antisense 5’-ATGGCAAGGGCAGAAGAA-3’ |
| Scara3(rat) | Nanjing jin si rui biotechnology co., LTD | sense 5’-ACCTACTGGGTTTCTATGGC-3’, antisense 5’-GTGTTGAGTGGGTTATTGC-3’ |
| actin(rat) | Nanjing jin si rui biotechnology co., LTD | sense 5’-CAAGAAGGAAGGCTGGAAAA-3’, antisense 5’-AGGGAAATCGTGCGTGAC-3’ |
| STAT1(rat) | Nanjing jin si rui biotechnology co., LTD | sense 5’-GCGACCAGAAACAGGAAC-3’, antisense 5’-CTGGAAGAGGACGAAGGT-3’ |
| Pou5f1(rat) | Nanjing jin si rui biotechnology co., LTD | sense 5’-CCTGGCTTCAGACTTCGC-3’, antisense 5’-TTCCACCTTCTCCAACTTCA-3’ |
| STAT3(rat) | Nanjing jin si rui biotechnology co., LTD | sense 5’-GCAGTATAGCCGATTCCT-3’, antisense 5’-TCTCCACCACTTTCATTTT-3’ |
| SP1(rat) | Nanjing jin si rui biotechnology co., LTD | sense 5’-GCAGTATAGCCGATTCCT-3’, antisense 5’-TCTCCACCACTTTCATTTT-3’ |
| SOX9(rat) | Nanjing jin si rui biotechnology co., LTD | sense 5’-GCCACCGAACAGACTCAC-3’, antisense 5’-GTTGGGCGGCAGGTATTG-3’ |
| TEAD1(rat) | Nanjing jin si rui biotechnology co., LTD | sense 5’-CTATCTATCCGCCCTGTGG-3’, antisense 5’-CCTGGCTGTCCTGTCTGTAT-3’ |
| **Chemicals, Enzymes and other reagents** |  |  |
| Gabapentin | Selleck | S2133 |
| 4-PBA | Santa Cruz Biotechnology | sc-200652 |
| ER-Tracker Red | Beyotime | C1041 |
| Bafilomycin A1(Baf-A1) | Selleck | S1413 |
| mRFP-GFP-LC3 adenovirus | Hanbio Biotechnology | HB-AP210 0001 |
| Nuclear and cytoplasmic protein extraction kit | Beyotime | P0028 |
| Protein A+G agarose | Beyotime | p2012 |
| Imprint® RNA immunoprecipitation kit | Sigma | RIP-12RXN |
| Dual-luciferase reporter gene assay kit | Beyotime | RG027 |
| **Software** |  |  |
| JASPAR2020 | http://jaspar.genereg.net/cart/ |  |
| LASAGNA-Search 2.0 | https://biogrid-lasagna.engr.uconn.edu/lasagna_search/ |  |
